# Nature of the optical band shapes in polymethine dyes and H-aggregates: dozy chaos and excitons. Comparison with dimers, H*- and J-aggregates

**DOI:** 10.1098/rsos.160550

**Published:** 2017-05-31

**Authors:** Vladimir V. Egorov

**Affiliations:** Photochemistry Center, Russian Academy of Sciences, 7a Novatorov Street, Moscow 119421, Russian Federation

**Keywords:** molecular quantum transitions, transient-state dynamics, dozy-chaos theory, optical band shapes, polymethine dyes, molecular aggregates

## Abstract

Results on the theoretical explanation of the shape of optical bands in polymethine dyes, their dimers and aggregates are summarized. The theoretical dependence of the shape of optical bands for the dye monomers in the vinylogous series in line with a change in the solvent polarity is considered. A simple physical (analytical) model of the shape of optical absorption bands in H-aggregates of polymethine dyes is developed based on taking the dozy-chaos dynamics of the transient state and the Frenkel exciton effect in the theory of molecular quantum transitions into account. As an example, the details of the experimental shape of one of the known H-bands are well reproduced by this analytical model under the assumption that the main optical chromophore of H-aggregates is a tetramer resulting from the two most probable processes of inelastic binary collisions in sequence: first, monomers between themselves, and then, between the resulting dimers. The obtained results indicate that in contrast with the compact structure of J-aggregates (brickwork structure), the structure of H-aggregates is not the compact pack-of-cards structure, as stated in the literature, but a loose alternate structure. Based on this theoretical model, a simple general (analytical) method for treating the more complex shapes of optical bands in polymethine dyes in comparison with the H-band under consideration is proposed. This method mirrors the physical process of molecular aggregates forming in liquid solutions: aggregates are generated in the most probable processes of inelastic multiple binary collisions between polymethine species generally differing in complexity. The results obtained are given against a background of the theoretical results on the shape of optical bands in polymethine dyes and their aggregates (dimers, H*- and J-aggregates) previously obtained by V.V.E.

## 1. Introduction

**Figure 1.**
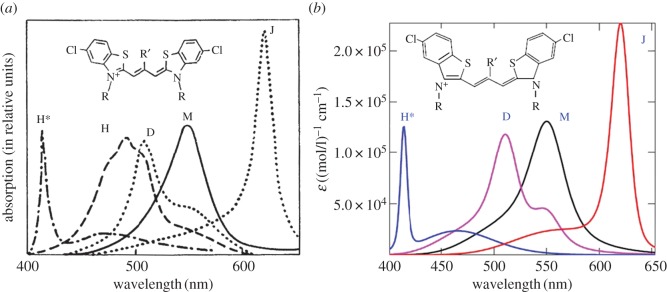
Theoretical optical absorption band shapes for thiapolymethinecyanine (*b*) fitted to the available experimental data on dye monomers (M) [3–5], their dimers (D) [6], J- [3–5] and H*-aggregates [7] (ε is the extinction coefficient) shown in the general picture ((*a*): H, H-aggregates) presented in 1977 [2].

Aggregation of polymethine dyes is one of the simplest and most striking examples of self-organization of organic matter at the supramolecular level. As a result of this self-organization, aggregates may occur, which vary in structure and in the number of molecules they contain, as can be seen from the variety of corresponding optical band shapes recorded in experiments [1,2]. Figure 1*a* shows a striking example [2] of such a diversity of optical band shapes for aggregates of polymethine dyes, which can be regarded as an experimental challenge to the theory [6,7]. Until very recently, these aggregates could not be observed directly in experiments, and optical spectroscopy methods were hence essentially the only way to study them. Although we have the opportunity today to observe aggregates (J-aggregates) using atomic force microscopy measurements [8], spectroscopic methods continue to be the main way to obtain information about them (e.g. [9–13] and the references therein).

Most simply, information about polymethine dyes and their aggregates can be obtained from the shape of the optical absorption bands. Here, we obtain information not so much about the aggregate as a whole but more about the optical chromophores that compose them. This information sometimes suffices to draw conclusions about the entire structure of the aggregate. (For example, the optical chromophore of J-aggregates, which consists of four monomers forming a brickwork-type structure, produces aggregates in the form of long thin rods [12,13]). The number of molecules in the optical chromophore and in the aggregate is often very different (as in the mentioned J-aggregate, for example). An obvious exception is the dimer, which is entirely an optical chromophore.

The most widely known aggregates of polymethine dyes are dimers and J-, H- and H*-aggregates (see the corresponding optical absorption band shapes in figure 1*a*). The absorption bands in the dimers and H- and H*-aggregates are blue-shifted relative to the monomer band. By contrast, the absorption band in J-aggregates is red-shifted. The bandwidth is increased when dimers and H-aggregates form and is significantly reduced when H*-aggregates form. The J-aggregation results in both a significant decrease in bandwidth and a considerable increase in intensity. This unique feature of J-aggregates is widely used in modern high technologies (e.g. [9,11–13] and the references therein).

Among experiments associated with measuring the shape of optical bands in polymethine dyes and their aggregates, experiments on measuring the shape of optical bands in concentration equilibria between monomers and dimers and between monomers and J-aggregates in aqueous solutions are distinguished [1,2]. These concentration equilibria are reversible and well controlled; the corresponding experiments serve both as a reliable source for producing dimers and J-aggregates in practice and as a good source of information about them [10,11]. The production of H- and H*-aggregates occurs in irreversible conditions [1,2], and these systems are less understood [9,11]. In this regard, any theoretical information about H- and H*-aggregates is of particular interest. Theoretical treatments of the optical band shapes for monomers (M) of polymethine dyes, their dimers (D) and J-aggregates (figure 1), as well as M–D and M–J concentration equilibria, were previously given by V.V.E. in Egorov [3–6] based on the so-called dozy-chaos theory of molecular quantum transitions (see comments below). The shape of the narrow optical band in H*-aggregates (figure 1) was treated by the V.V.E. in Egorov [7].

In this article, to clearly demonstrate the feasibility [14] of the dozy-chaos theory as applied to the optical band shapes in polymethine dyes and their aggregates [3–7,12,13], we discuss only one of many examples (but a striking one) of the theory and its applications, namely, the transferon resonance equation [3–5] and the relative intensity of the dozy-chaos optical bands in the vinylogous series of an ideal polymethine dye represented by thiapolymethinecyanine as solution-dependent [14]. We then theoretically interpret the shape of the optical absorption band in H-aggregates, which is shown in figure 1*a*. To highlight the features of the new results obtained here (§2: figure 6 and the related text about optical bands as solution-dependent; §3.4; §4 and §5), we present those features against a background of the theoretical results on the shape of optical bands in polymethine dyes and their aggregates (monomers, dimers, H*- and J-aggregates), which were previously obtained by V.V.E. (§2, except for figure 6 and the related text; §3, except for §3.4). For the self-sufficiency of this article, we briefly introduce the dozy-chaos theory on the qualitative (§2.1 and §2.2) and quantitative (§2.2) levels.

## 2. Dozy-chaos optical band shapes in monomers of polymethine dyes

### 2.1. Dozy chaos as a novel physical phenomenon in molecular quantum transitions

The fundamental distinction between molecules and free atoms is that atoms in a molecule are bound by electrons and oscillate. The fundamental difference between quantum (electron) transitions in molecules and quantum transitions in atoms is as follows: in a free atom, very light electrons are tightly bound to a single heavy nucleus and cannot control its motion in quantum transitions, but in a molecule, electrons are bound to multiple nuclei (at least two) and, by contrast, can control the motion of the nuclei in the processes of quantum transitions. As the saying goes, there is a reorganization of the nuclear motion. For example, as a result of an electronic excitation, the molecule becomes slightly swollen, and the nuclei in this excited state oscillate already about new equilibrium positions. The result of the reorganization of the nuclear motion is associated with a molecular reorganization energy, which can be calculated by quantum chemistry methods (e.g. [14] and the references therein). The question arises [12,13]: ‘How do light electrons in a very short**-**time quantum transition manage to control the motion of heavy nuclei having huge inertia?’ It turns out that before moving to an excited state, the electrons make the vibrational motion of the nuclei chaotic. As a result, the vibrational motion of the nuclei is partially transformed into their translational motion to new equilibrium positions. At the end of the quantum transition, because of chaos, the translational motion of the nuclei returns to vibrational motion but already about new equilibrium positions. This chaos is called dozy chaos [12,13], and it arises only during the short time of molecular quantum transitions. Dozy chaos emerges as a combined effect of the collective chaotic motion of electrons and nuclei and their chaotic electromagnetic interactions (dozy-chaos field or dozy-chaos radiation) in the transient state of molecules experiencing quantum transitions. Dozy chaos is a universal physical phenomenon, universal to the same extent that electrons and nuclei themselves are universal [6,7,15,16]. Dozy chaos is the main physical component in the dynamic self-organization of molecular quantum transitions. Because of dozy chaos, electron transitions in molecules are no longer quantum jumps between energy levels as occurs in atoms, and they have a continuous spectrum of electron-vibrational energies in the transient state. A molecule, being a quantum system in the ground and excited states, during a quantum transition thus becomes a classical system, which, as is well known, has a continuous energy spectrum [7]. This physical picture of molecular quantum transitions based on the concept of dozy chaos is confirmed by the successful qualitative and quantitative theoretical explanations of a large number of experimental data on the shape of the optical bands in polymethine dyes and their aggregates [3–7,12–17] and also many other experimental data related to elementary electron-charge transfers [6,18–22], which is the simplest case of molecular quantum transitions [6,15,16].

Formally, dozy chaos is introduced into the dynamics of molecular quantum transitions (elementary electron-charge transfers) by replacing the well-known infinitesimal imaginary addition *iγ* in the energy denominator of the total Green's function of the system of electrons and nuclei with a finite quantity [3–7,12,13,15,16] (see §2.2 below). The value of *γ* can be interpreted as the width of the vibronic energy levels arising in the transient state, but a detailed and successful comparison of the theory with experiment [3–7,12–14,16,17] shows that the value of *γ* significantly exceeds the energy gap between vibrational levels. Therefore, the idea of *γ* as the width of vibronic energy levels in the transient state is very rough. In fact, such a large value of *γ* indicates that the exchange of the kinds of motion and amounts of energy between electrons and nuclei in the transient state is so intense that it leads to chaos (dozy chaos) in their motion. On the other hand, such a large value of *γ*, leading to dozy chaos, points to the continuity of the spectrum of vibronic energy in the transient state, as mentioned above [7].

It is remarkable that in the case of strong dozy chaos (the gamma value is of the order of reorganization energy of the nuclear subsystem *E* in a quantum transition or even larger: *γ* *≥* *E*), the results of the new theory based on the Green's function method and dozy-chaos phenomenon are consistent with the results of the standard theory based on the Born–Oppenheimer adiabatic approximation [23] and the Franck–Condon principle [24–27] to a high degree of accuracy [4–7,12,13,16] (see §2.2 below). This is because the probabilities (per unit time) of quantum transitions in the case of strong dozy chaos no longer depend on the chaotic dynamics of the transient state and depend only on the initial and final states, which is also why dozy chaos so long eluded researchers' attention. Under the conditions of strong dozy chaos, which is often realized in practice, the standard theory of molecular quantum transitions hence gives correct results, although these results are obtained based on erroneous physical concepts [4–7,12,13,16].

In the case of very strong dozy chaos (γ≫E), the probabilities of quantum transitions are very small (in the classical language, we can speak about a very large internal friction in the system). The case of weak dozy chaos (γ≪E) corresponds to a high degree of dynamic self-organization of molecular quantum transitions and consequently to large transition probabilities [4–7,12,13,16] (see §2.2 below).

### 2.2. Dozy-chaos optical band shapes in elementary electron-charge transfers

#### 2.2.1. On the dozy-chaos theory of elementary electron-charge transfers

As compared with the Hamiltonian in the theory of many-phonon processes (see [28]), in the electrodynamics of extended multiphonon transitions [4] or, in other words, in the theory of elementary electron transfers, the Hamiltonian is only complicated by an extra electron potential well $V_{2}(\text{r}-\text{L})$ separated from the initial well $V_{1}(\text{r})$ by the length L≡|L|

2.1 $$H=-\frac{\hbar^{2}}{2m}\Delta_{\text{r}}+V_{1}(\text{r})+V_{2}(\text{r}-\text{L})+\sum_{\kappa }V_{\kappa }(\text{r})q_{\kappa }+\frac{1}{2}\sum_{\kappa }\hbar \omega_{\kappa }\left(q_{\kappa }^{2}-\frac{\partial^{2}}{\partial q_{\kappa }^{2}}\right),$$

where *m* is the effective electron mass, **r** is the electron's radius vector, *q_κ_* are the real normal phonon coordinates, *ω_κ_* are the eigenfrequencies of normal vibrations and *κ* is the phonon index; $\sum_{\kappa }V_{\kappa }(\text{r})q_{\kappa }$ is the electron–phonon interaction term.

Our intention is to find ‘good’ dynamic invariants for the transient state [5,29], which would be alternatives to the Born–Oppenheimer adiabatic invariants (the potential energy surfaces). Therefore, we seek the solution of the Schrödinger equation

2.2 $$H\Psi =E_{H}\Psi ,$$

for an ‘electron + environmental medium’ system using the Green's function procedure. At the start, we can regard identical conversions of the Schrödinger equation (2.2) in this procedure as alternatives to the identical conversions for extracting the non-adiabaticity operator in the Born–Oppenheimer procedure. The former must as far as possible retain the intercoupling of electronic and nuclear motions, while the latter are used to separate slow nuclear motion from fast electron motion.

The solution of equation (2.2) is written symbolically as [4,18]

2.3 $$\Psi_{1}=G_{H}\tilde{V}\Psi_{1}^{\mathrm{BO}},$$

where

2.4 $$G_{H}=G+G\tilde{V}G+G\tilde{V}G\tilde{V}G+\cdots ,$$

is the Green's function of Hamiltonian (2.1), and *G* is the Green's function of the Hamiltonian $H-\tilde{V}$

2.5 $$G(\text{r},{\text{r}}^{\prime };\;q,q^{\prime };\;E_{H}+\tilde{V})=\sum_{s}\frac{\Psi_{s}(\text{r},q){\Psi_{s}}^{*}({\text{r}}^{\prime },q^{\prime })}{E_{H}+\tilde{V}-E_{s}-i\gamma },$$

(spectral representation), with

2.6 $$\tilde{V}\equiv \sum_{\kappa }V_{\kappa }(\text{r})(q_{\kappa }-{\tilde{q}}_{\kappa }),$$

where ${\tilde{q}}_{\kappa }$ are the displacements of the normal phonon coordinates corresponding to the displacements of nuclear equilibrium positions in the medium, which are produced by the presence of an electron on donor 1 or acceptor 2. The superscript BO in equation (2.3) and hereafter means that the datum is taken in the adiabatic Born–Oppenheimer approximation.

The infinitesimal imaginary addition *iγ* is generally inserted when the Green's function is written in the spectral representation (see equation (2.5)) to avoid zero in the denominator (here, at $\tilde{V}(q=\tilde{q})=0$; see equation (2.6)). By contrast, we regard quantity *γ* as having a finite value, which gives it the physical sense of a measure of chaos in the environmental nuclear reorganization motion produced by electron movement from the donor to the acceptor. We call quantity *γ* the dozy-chaos energy [12,13]. In other words, the dozy-chaos energy *γ* characterizing the measure of chaos in the transient-state nuclear motion is introduced in the theory of elementary electron transfers, in addition to the nuclear reorganization energy $E\equiv \sum_{\kappa }\hbar \omega_{\kappa }{\tilde{q}}_{\kappa }^{2}/2$. Introducing the dozy-chaos energy *γ* allows avoiding a singularity in the probabilities of extended transitions (electron transfers) that follows from the incommensurability of masses of the electron and its environmental nuclei in the surrounding medium. Physically, it means introducing a mechanism in the transient state that first transforms part of the nuclear vibrational motion into translational motion and then returns the resulting translational motion into vibrational motion (see §2.1 above). Because chaos in the electron–nuclear motion develops only in the transient state and is absent in the initial and final states, it is called *dozy chaos*; hence, the corresponding energy *γ* is the *dozy-chaos energy*.

In addition to damping singular electron–nuclear motion, the postulate of the finite *γ* value implements one more indispensable function, namely, it gives the opportunity to introduce a small parameter into the theory [4,18]

2.7 $$\tilde{V}G∼\frac{\tilde{V}}{\gamma }∼\frac{\hbar \omega_{\kappa }}{\gamma }\ll 1.$$

It hence follows that

$$G\gg G\tilde{V}G\gg G\tilde{V}G\tilde{V}G\gg \ldots$$

and, according to equation (2.4), we obtain

2.8 $$G_{H}\approx G.$$

In accordance with equation (2.3), we thus obtain the solution that describes the electron-transfer state

2.9 $$\Psi_{1}\approx G\tilde{V}\Psi_{1}^{\mathrm{BO}},$$

where $G=G(E_{H}=E_{H}^{\mathrm{BO}};\;i\gamma ,\gamma \gg \hbar \omega_{\kappa })$.

It is evident from equation (2.7) that smallness in the transition amplitude

2.10 $$A_{12}=\langle \Psi_{2}(\text{r}-\text{L},q)|V|\Psi_{1}(\text{r},q)\rangle$$

can be avoided if the system's wave function for an electron localized on acceptor 2 must be taken not in form (2.9) but in the adiabatic approximation:^1^ $\Psi_{2}=\Psi_{2}^{\mathrm{BO}}$.

By virtue of equation (2.7), the series for the transition probability, corresponding to series (2.4) for the Green's function *G*_H_, has a small parameter ${\left({\bar{n}}_{1}\hbar \omega_{\kappa }/\gamma \right)}^{2}\ll 1$, where ${\bar{n}}_{1}$ is the Planck distribution function.^2^ Hence, the small parameter of the problem for $k_{B}T>\hbar \omega_{\kappa }/2$ (*T* is the absolute temperature) is given by

2.11 $${\left(\frac{k_{B}T}{\gamma }\right)}^{2}\ll 1.$$

Proceeding in accordance with the rules of quantum mechanics (in the framework of the ‘Fermi golden rule’) and using the method first described in Egorov [31,32], which generalizes the generating polynomial method of Krivoglaz & Pekar [33,34] in the theory of many-phonon processes [28], we obtain the general expression for the probabilities of electron phototransfers (for an optical absorption *K*) [4,18]. The calculations [4,18] are simplified by applying the Fermi zero-range approximation [35,36] for electron potential wells.

#### 2.2.2. Result

Based on the general expression for the optical absorption *K* and applying exact methods of the theory of functions of complex variables, within the framework of the Einstein nuclear vibration model, we obtain an analytical result completely expressed in elementary functions [3,4,18]:

2.12 K=K0 exp⁡W,

2.13 $$\begin{array}{rl} W & =\frac{1}{2}\ln\left(\frac{\omega \tau \sinh\;\beta_{T}}{4\pi \cosh\;t}\right)-\frac{2}{\omega \tau }\left(\coth\beta_{T}-\frac{\cosh\;t}{\sinh\;\beta_{T}}\right)+(\beta_{T}-t)\frac{1}{\omega \tau \Theta }-\frac{\sinh\;\beta_{T}}{4\omega \tau \Theta^{2}\cosh\;t}, \end{array}$$

2.14 $$\begin{array}{rl} 1 & \ll \frac{1}{\omega \tau \Theta }\leq \frac{2\cosh\;t}{\omega \tau \sinh\;\beta_{T}}, \end{array}$$

where $\beta_{T}\equiv \hbar \omega /2k_{B}T$ (*ω* is the phonon frequency) and

2.15 $$\begin{array}{rl} t & =\frac{\omega \tau_{e}}{\theta }\left[\frac{AC+BD}{A^{2}+B^{2}}+\frac{2\Theta (\Theta -1)}{{\left(\Theta -1\right)}^{2}+{\left(\Theta /\theta_{0}\right)}^{2}}+\frac{{\theta_{0}}^{2}}{{\theta_{0}}^{2}+1}\right], \end{array}$$

2.16 $$\begin{array}{rl} |\theta_{0}| & \gg \frac{E}{2J_{1}}, \end{array}$$

2.17 $$\begin{array}{rl} \theta & \equiv \frac{\tau_{e}}{\tau }=\frac{LE}{\hbar \sqrt{2J_{1}/m}},\;\Theta \equiv \frac{\tau^{\prime }}{\tau }=\frac{E}{\Delta },\;\theta_{0}\equiv \frac{\tau_{0}}{\tau }=\frac{E}{\gamma } \end{array}$$

2.18 $$\begin{array}{rl} \mathrm{and}\;\tau_{e} & =\frac{L}{\sqrt{2J_{1}/m}},\;\tau =\frac{\hbar }{E},\;\tau^{\prime }=\frac{\hbar }{\Delta },\;\tau_{0}=\frac{\hbar }{\gamma }. \end{array}$$

Here, we use the notation

2.19 $$\begin{array}{rl} & \begin{array}{rl} A & =\cos\left(\frac{\theta }{\theta_{0}}\right)+\Lambda +{\left(\frac{1}{\theta_{0}}\right)}^{2}N,\\ B & =\sin\left(\frac{\theta }{\theta_{0}}\right)+\frac{1}{\theta_{0}}M, \end{array}\} \end{array}$$

2.20 $$\begin{array}{rl} & C=\theta \left[\cos\left(\frac{\theta }{\theta_{0}}\right)-\frac{1-\xi^{2}}{2\theta_{0}}\sin\left(\frac{\theta }{\theta_{0}}\right)\right]+M, \end{array}$$

2.21 $$\begin{array}{rl} & D=\theta \left[\sin\left(\frac{\theta }{\theta_{0}}\right)+\frac{1-\xi^{2}}{2\theta_{0}}\cos\left(\frac{\theta }{\theta_{0}}\right)\right]-\frac{2}{\theta_{0}}N, \end{array}$$

2.22 $$\begin{array}{rl} \mathrm{and}\;\xi & \equiv {\left(1-\frac{E}{J_{1}}\right)}^{1/2}\;(J_{1}>E\;\mathrm{by}\;\mathrm{definition}), \end{array}$$

and where we finally have

2.23 $$\begin{array}{rl} \Lambda & =-{\left(\Theta -1\right)}^{2}E+\left[\frac{(\Theta -1)\theta }{\rho }+\Theta (\Theta -2)\right]E^{1-\rho /1-\xi }, \end{array}$$

2.24 $$\begin{array}{rl} M & =2\Theta (\Theta -1)E-\left[\frac{(2\Theta -1)\theta }{\rho }+2\Theta (\Theta -1)\right]E^{1-\rho /1-\xi }, \end{array}$$

2.25 $$\begin{array}{rl} N & =\Theta \left[\Theta E-\left(\frac{\theta }{\rho }+\Theta \right)E^{1-\rho /1-\xi }\right] \end{array}$$

2.26 $$\begin{array}{rl} \mathrm{and}\;E & \equiv \exp\left(\frac{2\theta }{1+\xi }\right)\;\rho \equiv \sqrt{\xi^{2}+\frac{1-\xi^{2}}{\Theta }}. \end{array}$$

The factor *K*_0_ becomes

2.27 $$K_{0}=K_{0}^{e}K_{0}^{p},$$

where

2.28 $$K_{0}^{e}=\frac{2\tau^{3}J_{1}}{m}\frac{(A^{2}+B^{2})\rho^{3}\Theta^{4}\xi }{\theta^{2}{\left[{\left(\Theta -1\right)}^{2}+{\left(\Theta /\theta_{0}\right)}^{2}\right]}^{2}[1+{\left(1/\theta_{0}\right)}^{2}]}\exp\left(-\frac{4\theta }{1-\xi^{2}}\right)$$

and

2.29 $$K_{0}^{p}=\frac{1}{\omega \tau }{\left[1+\frac{\sinh(\beta_{T}-2t)}{\sinh\;\beta_{T}}\right]}^{2}+\frac{\cosh(\beta_{T}-2t)}{\sinh\;\beta_{T}}.$$

Inequalities (2.14) and (2.16) are not any significant restrictions on the system parameters and are associated with items of routine approximations made in the calculations [4,18], the discussion of which is beyond the scope of this article. In equations (2.17) and (2.18), *J*_1_ is the electron binding energy in the initial state 1, and *Δ* defines the thermal effect energy (the heat energy) in elementary electron-transfer processes (extended quantum transitions or extended multiphonon transitions). The energy ℏΩ of the absorbed photon and the heat energy *Δ* > 0 are related by the law of conservation of energy^3^

2.30 $$\hbar \Omega =J_{1}-J_{2}+\Delta ,$$

where *J*_2_ is the electron binding energy in the final state. The wavelength *λ*, indicated on the *x*-axis in the figures below, is related to the frequency *Ω* in equation (2.30) by the standard formula $\lambda =2\pi c/\Omega n_{\mathrm{ref}}$ (*c* and *n*_ref_ are the speed of light in vacuum and the refractive index). The time scales given by equations (2.18) control the dynamics of extended quantum transitions. They are discussed in detail elsewhere [5,12,13,18,29]. We discuss them briefly here.

An important element in the dynamics of extended quantum transitions is the quantitative relation between the time

2.31 $$\tau_{e}=\frac{L}{\sqrt{2J_{1}/m}}$$

and the time

2.32 $$\tau =\frac{\hbar }{E},$$

which are part of the above dozy-chaos equations for calculating optical absorption band shapes (see equations (2.18)). The time *τ*_e_ is the characteristic time of electron motion in the donor–acceptor system; the time *τ* is the characteristic time of reorganization of the nuclear subsystem. In the case 2*τ*_e_ *=* *τ*, we have the so-called transferon resonance [5] between the characteristic frequency ${\left(2\tau_{e}\right)}^{-1}$ of an extended electron motion in the system of the donor and the acceptor, separated by distance *L*, and the characteristic frequency $\tau^{-1}$ of the motion of the reorganization of the nuclei of a medium in which the donor and the acceptor are embedded. We consider one of the implications of the discussed resonance

2.33 $${\left(2\tau_{e}\right)}^{-1}=\tau^{-1}$$

as an example in §2.4 below. Further, in equations (2.18), the time $\tau^{\prime }=\hbar /\Delta$ is the characteristic time of conversion of the light energy ℏΩ into the electron excitation energy *J*_1_−*J*_2_ and the heat energy *Δ* in elementary electron-transfer processes (see equation (2.30)), and the time $\tau_{0}=\hbar /\gamma$ is the characteristic time of conversion of electron motion (energy) into nuclear reorganization motion (energy) (*γ* > 0) or/and of the inverse processes (*γ* < 0) in the chaotic transient state (see below about the sign of *γ*). The dimensionless parameters *θ*, *Θ* and *θ*_0_ (see equations (2.17)) associated with the above characteristic times *τ*_e_, *τ′* and *τ*_0_ are introduced relative to the characteristic time *τ* (equation (2.32)). The quantities Λ=Λ(θ,Θ), M=M(θ,Θ) and N=N(θ,Θ) (see equations (2.23)–(2.25)) are independent of the parameter *θ*_0_ (i.e. are independent of the dozy-chaos energy *γ*), and therefore do not include the process of chaotization of motion of the electron and nuclei in the transient state and correspond only to their regular movement. The quantities $A=A(\theta ,\Theta ,\theta_{0})$, $B=B(\theta ,\Theta ,\theta_{0})$, $C=C(\theta ,\Theta ,\theta_{0})$ and $D=D(\theta ,\Theta ,\theta_{0})$ (see equations (2.19)–(2.21)), which depend on the parameter *θ*_0_, already include the process of chaotization of motions of the electron and nuclei in the transient state. The equations, which do not include the parameter $\beta_{T}\equiv \hbar \omega /2k_{B}T$ (i.e. do not include the absolute temperature *T*; see equations (2.15)–(2.26), (2.28)), correspond to the dynamics of the transient state in the ensemble of the donor–acceptor systems without taking the averaging over the equilibrium distribution of their initial states into account. By contrast, the equations that include the parameter $\beta_{T}\equiv \hbar \omega /2k_{B}T$ (see equations (2.13) and (2.29)) correspond to the dynamics of the transient state in the ensemble of the donor–acceptor systems with the averaging over the equilibrium distribution of their initial states already taken into account. Each donor–acceptor system in the ensemble has its value of the parameter *Θ* (i.e. its value of the thermal effect *Δ* and hence its value of the frequency *Ω* of the absorption of light; see equation (2.30)). The entire set of values of the parameter *Θ* in the ensemble and hence of values of the frequency *Ω*, together with the other system parameters, determines the position, width, intensity and shape of optical bands. It follows from the formula for the characteristic time of conversion of the light energy ℏΩ, $\tau^{\prime }=\hbar /\Delta$ (see equations (2.18) and (2.30)) that the dynamics of producing the shape of the optical bands is most rapid in the high-frequency wing of the optical bands and is slowest in their low-frequency wing. Finally, in equation (2.28), $\exp[-4\theta /(1-\xi^{2})]\equiv \exp(-2L/a)$ is the Gamow tunnel factor ($a\equiv \hbar /\sqrt{2mJ_{1}}$).

The quantity $K=K(\Theta ,\theta_{0})$ (see equation (2.12)) and the corresponding optical extinction

2.34 $$\varepsilon =\frac{4\pi^{2}q^{2}N_{A}\Omega }{3\hbar cn_{\mathrm{ref}}}K$$

(*q* is the amount of electron charge transferred in an extended multiphonon transition, and *N*_A_ is the Avogadro constant) have a singularity at the point $\left(\Theta =1,\theta_{0}=\infty \right)$ or (Δ=E,γ=0). The character of this singularity is determined by singularities of the functions $t=t(\Theta ,\theta_{0})$ in equation (2.15) and $K_{0}^{e}=K_{0}^{e}(\Theta ,\theta_{0})$ in equation (2.28). The singularity in the function $K_{0}^{e}=K_{0}^{e}(\Theta ,\theta_{0})$ is removable:

2.35 K0e(Θ=1,θ0→∞)2τ3J1/m=ξθ2[ exp (2θ1+ξ)−θ22−θ−1]2 exp (−4θ1−ξ2).

**Figure 2.**
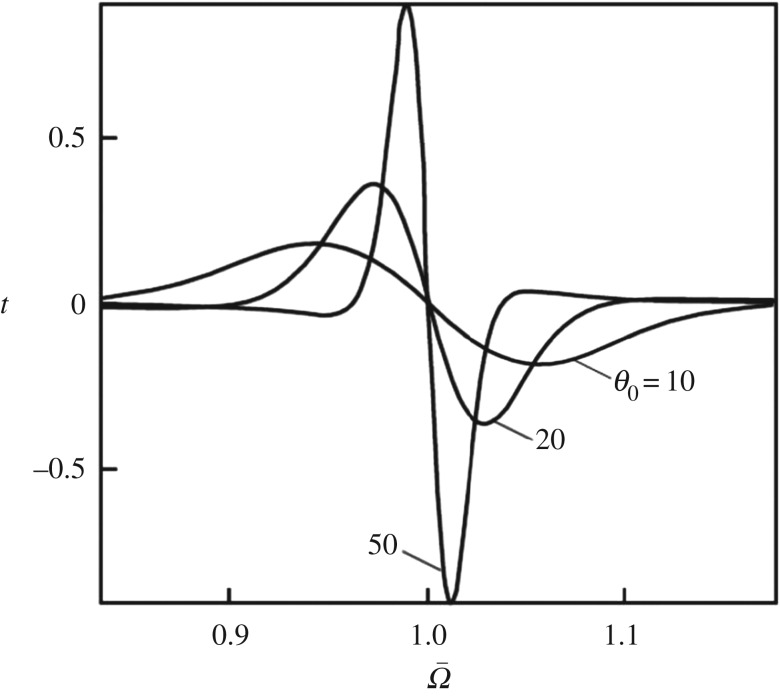
The electrodynamics of extended multiphonon transitions in the vicinity of the singular point $\left(\bar{\Omega }=1,\theta_{0}=\infty \right)$ is illustrated by the behaviour of the function $t=t(\bar{\Omega },\theta_{0})$ (see equation (2.15)) [4]. For simplicity here, we formally assumed that *J*_2_−*J*_1_ = 0. Then $\Theta^{-1}=\hbar \Omega /E\equiv \bar{\Omega }$. For the ‘electron + environment’ system, we used the parameters *J*_1_ = 5 eV, *E* = 1 eV, *m* = *m*_e_, *ω* = 5 × 10^13^ s^−1^ and *L* = L* ≈ 0.44 nm (transferon resonance, see equation (2.33)). Reproduced from Egorov [4] by permission of Elsevier, © 2001.

In the function $t=t(\Theta ,\theta_{0})$, the singularity at the point $\left(\Theta =1,\theta_{0}=\infty \right)$ is non-removable. The behaviour of the function $t=t(\Theta ,\theta_{0})$ in the vicinity of $\left(\bar{\Omega }\equiv \Theta^{-1}=1,\theta_{0}=\infty \right)$ is illustrated in figure 2. We note that the result (2.12)–(2.29) is invariant under changing the sign of *γ*. This invariance is in line with the physical fact that both the virtual processes of conversion of electron motion (energy) into nuclear reorganization motion (energy) and the inverse processes take place in the chaotic transient state of the ‘electron + nuclear environment’ system [18]. For definiteness, we set *γ* > 0 in figure 2 and hereinafter.

#### 2.2.3. Passage to the standard result

**Figure 3.**
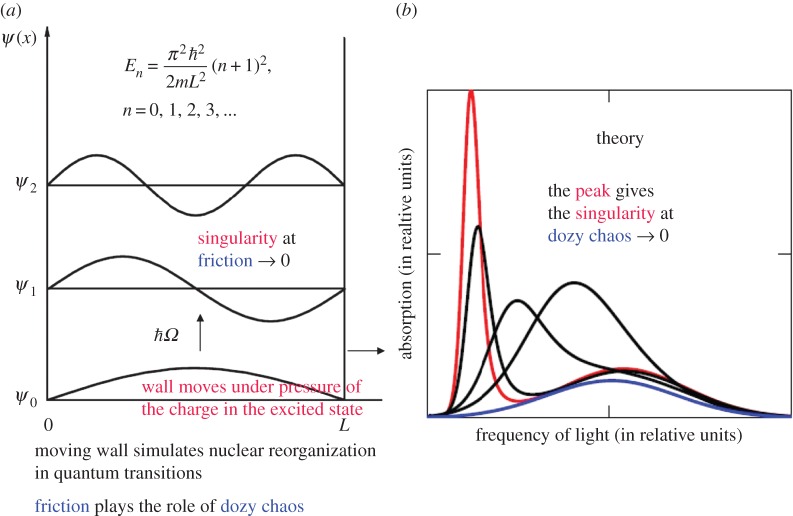
Singularity in the probability of molecular quantum transitions: a potential box with a movable wall (*a*) and the optical absorption band shape dependent on the dozy chaos available to a given quantum transition (*b*); the band shape with the strongly pronounced peak (J-band) corresponds to the least dozy chaos [6]. (Original citation)—Reproduced by permission of The Royal Society of Chemistry.

The limit passage from expressions (2.12)–(2.30) for the optical absorption *K* to the standard result in the theory of many-phonon processes [28] could be realized by letting the dozy-chaos energy *γ* tend to either zero or infinity, but *K* is infinite in the first case and vanishes in the second. The physical sense of K(γ→0)→∞ beyond the adiabatic approximation relates to the incommensurability of masses of the electron and its environmental nuclei in the surrounding medium (see §2.2.1 above). The physical sense of K(γ→∞)→0 (see §2.1 above and figure 3 below) is predetermined by the impossibility of electronic quantum transition coupled to nuclear reorganization at absolutely chaotic (random) movements of nuclei in the transient state (i.e. at infinite ‘friction’ in the electron–nuclear system). Given this, we can eliminate *γ* in expressions (2.12)–(2.30) and obtain the standard result by letting *γ* tend to infinity in the expression for *t* (*t* → 0; figure 2, where $\theta_{0}=E/\gamma$ according to equation (2.17)) and to zero in $K_{0}^{e}$ (see equation (2.35)). As a result, we obtain an equation of the standard type for the optical absorption *K* (for $k_{B}T>\hbar \omega /2$) [4]

2.36 K=a2ℏ4πλrkBT exp (−2La) exp [−(Δ−λr)24λrkBT],

where $\lambda_{r}\equiv 2E$.

#### 2.2.4. A potential box with a movable wall and the optical absorption band shape dependent on the dozy chaos available to a given quantum transition

In a simple style, the singularity in the probabilities of electron–nuclear(–vibrational) transitions can be illustrated by a potential box with a movable wall (figure 3*a*) [6,12,13]. The wall is fastened to the abscissa axis by a freely movable joint and can move with a certain friction or without friction against the axis. Such a wall simulates the environmental nuclear reorganization in the molecular quantum transitions, where dozy chaos plays the role of friction. In the theory [3–6,12,13,18,29], this results in the dozy-chaos dependent optical absorption band being displaced to the red spectral region and narrowed (figure 3*b*). As can be seen from figures 2 and 3, the intensity and the width of the optical band are determined by the ratio between the dozy-chaos energy *γ* and the reorganization energy *E*. The smaller the value of *γ* is, the more ‘splash’ in the dynamic function $t=t(\bar{\Omega },\theta_{0})$ ( figure 2, $\theta_{0}\gg 1$ or γ≪E), the higher the degree of organization of the molecular quantum transition, and the more the intensity and less the width of the optical band (figure 3). The red shift of the peak in figure 3*b* can be easily conceived from the potential box with a movable wall (figure 3*a*). This can be also understood from the behaviour of the band shape when the nuclear reorganization energy changes (decreases) in the standard theory, if we regard the reorganization energy as a complex value whose imaginary part is the dozy-chaos energy *γ* (see details in [6]).

### 2.3. Polymethine chain as the optical electron-transfer chromophore in monomers of polymethine dyes

**Figure 4.**
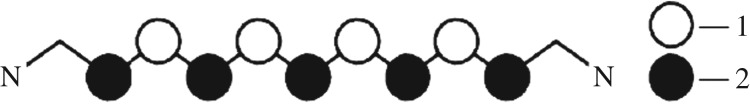
Ideal polymethine state [37,38]. Charges reside on carbon atoms of the polymethine chain in the ground state; charges: 1, positive; 2, negative [6]. (Original citation)—Reproduced by permission of The Royal Society of Chemistry.

The simplest example of the considered molecular quantum transitions with the dynamics of their transient states taken into account is the quantum transitions in the main optical chromophore of polymethine dyes placed in a solvent—in the system ‘polymethine chain + environment’. According to the concept of the so-called ideal polymethine state, formulated by Dähne [37], the main element of a chromophore in the polymethine dyes has a markedly extended *π*-electron-charge density. It strongly alternates along the quasi-linear polymethine chain and is alternately redistributed on optical excitation (figure 4) [38]. For the first excited state, the moment of electron transition is directed along the polymethine chain [38]. This corresponds to the elementary electron-charge transfer along the chain [3–6,12,13]. Therefore, in equations (2.17) and (2.18), the distance *L* can be regarded as the polymethine chain length. The polymethine dyes discussed below belong to the ideal polymethine state considered.

In polymethine dyes, a complete charge transfer along the entire length *L* of the polymethine chain is the sum of many acts of transfer of a relatively small number of charges over the small distance between neighbouring carbon atoms (figure 4). Therefore, the tunnel effects are small, and formally replacing a large number with *η* ≤ 1 for the Gamow tunnel factor $\exp[-4\theta /(1-\xi^{2})]\equiv \exp(-2L/a)$ in equation (2.28) [4,5] reduces the problem of the transfer of an alternating charge along the polymethine chain to that of electron transfer.

For polymethine dyes, by their specific structure (the sufficiently long linear polymethine chain is their main chromophore), the number of environmental nuclei tightly coupled to the electron transition in a molecule considerably exceeds the number of corresponding nuclei in a molecule itself. Therefore, we neglect nuclear motion in the transient state in a molecule itself. A similar statement is true for dye aggregates. Hence, the interaction of electron transitions with the environment is the controlling factor in the design of the physical picture of quantum transitions in polymethine dyes [3–6,12,13].

### 2.4. Resonance nature of the shapes of the dozy-chaos optical bands as solution-dependent in the vinylogous series of thiapolymethinecyanine

#### 2.4.1. Inconsistency of applying the standard electron-transfer theory to electron-charge transfers in a polymethine dye chromophore

As mentioned above, the problem of alternating charge transfer along the main chromophore of a polymethine dye (polymethine chain; figure 4) reduces to that of elementary electron transfer by formally replacing a large number with *η* ≤ 1 for the Gamow tunnel factor [3–6,12,13].

**Figure 5.**
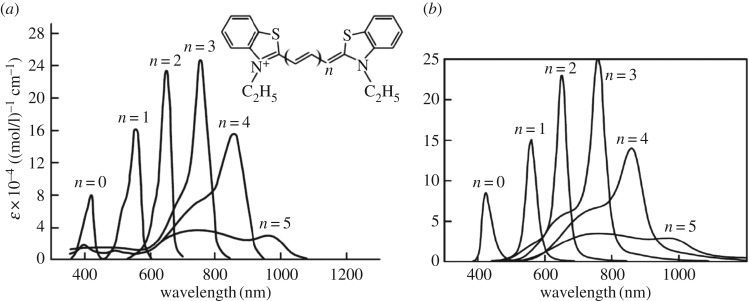
Experimental [2,39] (*a*) and theoretical [5] (*b*) monomer's optical absorption band shapes dependent on the length of the polymethine chain (thiapolymethinecyanine in methanol at room temperature) [6]. (Original citation)—Reproduced by permission of The Royal Society of Chemistry. For short chains (*n* = 0, 1, 2, 3), tunnel effects can be neglected (*η* = 1). For long chains (*n* = 4,5), although the tunnel effects are small, they must be taken into account (*η* < 1). Also see the caption to figure 6 below.

We consider the most intense optical absorption band in the known Brooker series [2,39] (figure 5) corresponding to the polymethine chain length *L* = 1.4 nm. The band is first treated based on the result of the standard electron-transfer theory for the optical absorption *K* (for $k_{B}T>\hbar \omega /2$) [28,33,34,40–52]

2.37 $$K\propto \exp\left[-\frac{{\left(\Delta -\lambda_{r}\right)}^{2}}{4\lambda_{r}k_{B}T}\right],$$

(cf*.* equation (2.36)), where thermal effect *Δ* is obtained from equation (2.30). The band half-width

2.38 w1/2=22 ln⁡22λrkBT≈0.09 eV

(*T* = 300 K) is used to estimate the Marcus energy $\lambda_{r}\equiv 2E$ of the environmental nuclear reorganization. The result is *λ*_r_ ≈ 0.03 eV. The standard theory gives the Gaussian function for the band shape (equation (2.37)). Therefore, it does not explain the explicitly asymmetric band shape observed in experiment. It was supposed that the explanation could be deduced from the new theory [4,5] (see the result in §2.2.2.) on the assumption that the most intense band in the Brooker series corresponds to the transferon resonance (see equation (2.33)). From the transferon resonance, it turns out that *λ*_r_ ≈ 0.63 eV (see equation (2.31), where *L* = 1.4 nm and J≅5 eV). Hence, this reorganization energy is 21 times (**!**) that in the standard theory [5]. The value of the reorganization energy [53–57] was obtained by V.V.E. in 2002 [5] by the above estimation using the simple resonance equation (equation (2.33)) and was recently supported by quantum-chemical calculations [14].

The standard approach grossly underestimates nuclear reorganization energy because it is based on the Franck–Condon principle [24–27], which treats electron transitions as instantaneous. Therefore, applying the standard theory to an extended system such as a polymethine dye leads to some local, but not total, environmental nuclear reorganization energy [5].

#### 2.4.2. Nature of the shape of a polymethine dye optical band: the charge-transfer effect with regard for the chaotic character of the environmental nuclear reorganization. Explaining the experimental data of Brooker and co-workers

**Figure 6.**
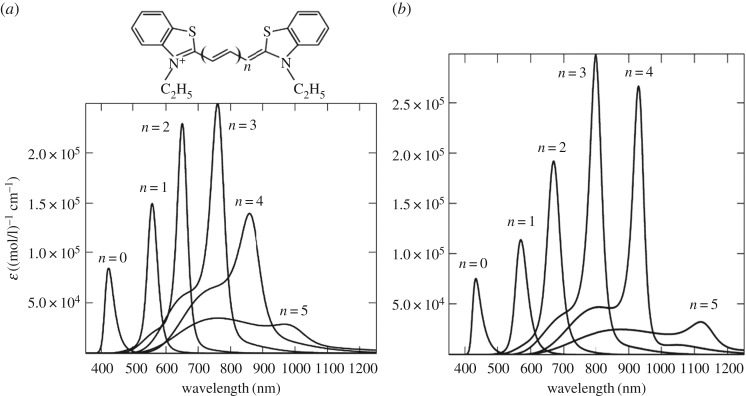
Theoretical dependence of optical absorption of an ideal polymethine dye (thiapolymethinecyanine) [5] on the polymethine chain length 2(*n* + 2)*d*, where *d* are certain roughly equal bond lengths in the chain. In (*a*), the absorption bands are computed using equations (2.12)–(2.30) and (2.34) with *η* ≤ 1 instead of the Gamow tunnel factor [3–6,12,13] when fitting them to experimental data of Brooker and co-workers (figure 5*a*) in terms of the wavelength *λ*_max_, extinction ε_max_, and half-width *w*_1/2_ with a high degree of accuracy. We used the ‘dye + environment’ system parameters [5] *q* = *e*, where *e* is the electron charge, *m* = *m*_e_, *ω* = 5 × 10^13^ s^−1^, *d* = 0.14 nm, *n*_ref_ = 1.33; for *n* = 0,1,2,3,4,5 we respectively have *J*_1_ = (5.63, 5.40, 4.25, 3.90, 3.74, 3.40) eV, *J*_1_−*J*_2_ = (1.71, 1.31, 1.11, 0.90, 0.74, 0.40) eV, *E* = (0.245, 0.248, 0.256, 0.275, 0.297, 0.496) eV, and *γ* = (0.402, 0.205, 0.139, 0.120, 0.129, 0.131) eV; the factor *η* = 1 for *n* = 0,1,2,3 and *η* = 0.55,0.1 respectively for *n* = 4,5 (also see the caption to figure 5 above); *T* = 298 K. In (*b*), *E*_right_ = 0.8*E* and $\gamma_{\mathrm{right}}=(0.402,0.205\times 1.2,\;0.139\times 1.2,0.120/1.1,0.129/1.8,0.131/1.5)$, the other parameters are the same as in (*a*).

Figures 5*b* and 6*a* present the results of fitting [5] our theoretical extinction (2.12)–(2.30), (2.34) to Brooker's experimental data [2,39] ( figure 5*a*). The fitting was realized in terms of the maximum position, maximum intensity and band half-width. The theoretical band shape proved to be very similar to the experimental one.

The appropriate choice of system parameter values in the theoretical equations was possible because the assumption in the above section proved justified; indeed, the most intense band in the Brooker series corresponds to the transferon resonance (2.33). It allows using the polymethine chain length to estimate the nuclear reorganization energy of the environment (methanol) [5].

The successful explanation of Brooker's experimental data, based on our theoretical equations (2.12)–(2.30), (2.34) for the band shape, can be attributed to the fact that the new charge-transfer theory [4,6,12,13], in which these equations were derived, accounts for the chaotic nature of the dynamics of the environmental nuclear reorganization in the transient state of elementary charge transfers—a simple particular case of molecular quantum transitions.

#### 2.4.3. Resonance in the series of dozy-chaos optical bands as solution-dependent

The resonance nature of the shape of the dozy-chaos optical bands in the vinylogous series of thiapolymethinecyanine is associated with the transferon resonance (equation (2.33)) and was first demonstrated by V.V.E. in 2002 [5] (also see [12,13]). The change in the value of the reorganization energy *E* in the resonance equation (equation (2.33)) leads to a change in the value of the resonance length *L* of the polymethine chain. In this regard, we ask: ‘How does the picture of bands in the vinylogous series of thiapolymethinecyanine change, if we replace one solvent with another, for example, a high-polarity solvent (in our case, methanol) with a less polar solvent?’ In our case ( figure 6*a*), the resonance is near the chain length *n* = 3. Reducing the solvent polarity obviously shifts this resonance toward the chain lengths *n* = 4 and *n* = 5. Figure 6*b* shows the transformation of the picture of the band shapes shown in figure 6*a* as a result of reducing the reorganization energy *E*. This takes into account that near a transferon resonance, the dynamics of the transient state is more organized and hence less chaotic, which corresponds to a reduced value [5] of the dozy-chaos energy *γ* (also see [12,13]). Comparing figure 6*b* versus figure 6*a*, we can see that replacing a polar solvent with a less polar solvent leads to changing the sign of the relative intensity of the bands for the chain lengths *n* = 2 and *n* = 4, which are closest to the resonance band (*n* = 3). Using our theory and the experimental data from Kachkovski *et al.* [58], this effect was first noted in Petrenko & Stein [14] in the two cases where: (i) the transferon resonance in a dye series (thiacyanines) is at *n* = 3 (compare figure 6 (*n* = 2 and *n* = 4) from this article and fig. 2 (labels 2 and 4) in [58]), and (ii) the transferon resonance in a dye series (thiapyrylocyanines) is at *n* = 2 (in [58], compare fig. 4 (labels 2 and 4) and fig. 3 (labels 2 and 4)).

## 3. Dozy-chaos optical band shape in H-aggregates compared with the shape in J-aggregates, dimers and H*-aggregates

### 3.1. Dozy-chaos narrow J-band and pi-stacking in the J-aggregate chromophore

**Figure 7.**
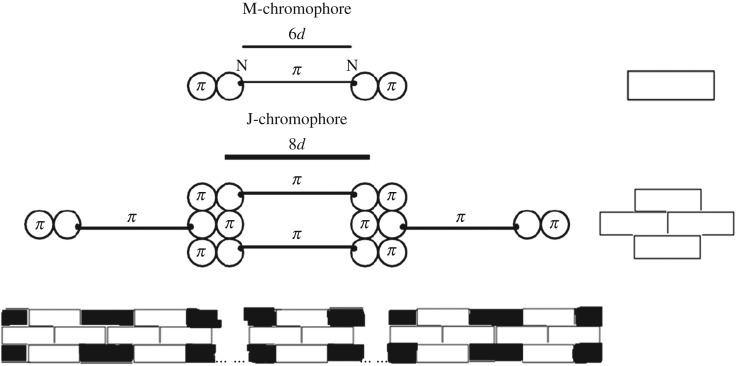
A polymethine dye molecule and its brickwork-structure J-aggregate [1,2,59]. Dye chromophore lengthening is a result of J-aggregation through *π*–*π* electron interaction between the polymethine chain and the heterocyclic rings, where *d* is a certain unitary bond length in each chromophore [3,4].

The nature of the narrow red-shifted J-band (figure 1) is explained by a good dynamic self-organization of the quantum transitions in the chromophore of J-aggregates for which the environmental nuclear dynamics in weak dozy chaos contributes to the electronic transition in the J-chromophore [3–5,12,13]. The chromophore of J-aggregates is composed of four molecules forming a brickwork-type structure [3–5,12,13]. The brickwork structure [1,2,59] of the J-chromophore is formed by stacking the pi-system of the polymethine chain of one molecule with the pi-systems of benzene rings of two neighbouring molecules (figure 7) [3,4]. The fourth molecule, fixing the brickwork, is assumed to be ‘neutral’ in relation to the pi-stacking in the other three molecules. We note that the now extremely popular idea of pi-stacking, used in the design of various organic molecular systems [60], was expressed in 2001 in relation to the structure of J-aggregates by V.V.E. [3,4] and the nature of pi–pi interactions was first clearly considered in 1990 by Hunter & Sanders [61]. The nature of pi-stacking is currently being investigated actively using advanced computational methods of quantum chemistry [62–67].

As mentioned above, in the J-aggregates, four dye molecules form chromophores of the brickwork structure as thick as three bricks. These chromophores organize J-aggregates that look like long thin rods (figure 7, bottom) [3–5,12,13]. Such a structure minimizes the potential parasitic exciton effects between the J-aggregate chromophores on the optical band shape [3–5,12,13], and exciton effects on the shape of the J-band are hence negligible [12,13].

### 3.2. Dozy-chaos–exciton coupling in dimers

In dimers of polymethine dyes in contrast with J-aggregates, the exciton effects turn out to be essential in the dynamics of molecular quantum transitions, and the pure dozy-chaos dynamics of quantum transitions in their constituent monomers is also essential [12,13]. The exciton in them is defined as the Frenkel exciton [68,69], which can be conveniently interpreted as an electron-excited molecular state passing from one site to another.

**Figure 8.**
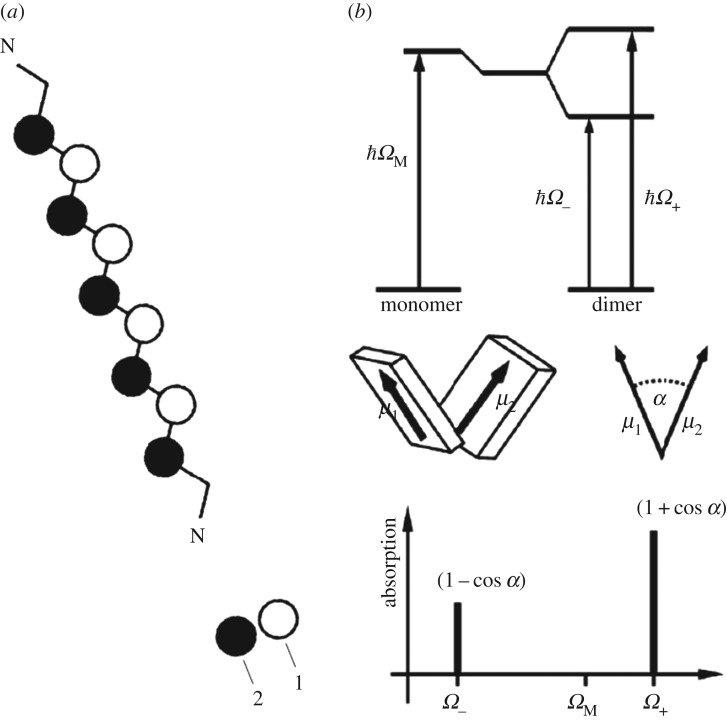
Ideal polymethine state (*a*) [37,38] shown above (figure 4). Excitonic shift and splitting in molecular dimers for an alternate orientation of transition dipole moments (*b*) [68–70]. When the interaction of both exciton transitions with the environment is absent, the optical absorption spectrum is given by two zero-width lines shifted relative to one another along the frequency axis (bottom right). ‘Switching on’ this interaction results in the transformation of both zero-width lines to optical bands of a non-zero width. The optical band shape for the dimers of a polymethine dye is obtained [6,7] by determining the interaction of electron (exciton) transitions with the motion of nuclei of the environment in the framework of the dozy-chaos theory of molecular quantum transitions [6].

In dimers, intermolecular interaction results in the shift of the molecular excitation energy level of the monomer (excitonic shift) and its splitting (excitonic splitting), shown in figure 8. Knowing the transition dipole moments of the constituent molecules, their mutual orientation, and the intermolecular distance allows calculating the excitonic shift and splitting of the dimer. The oscillator strengths of radiative transitions to the lower and upper states depend on the angle between the transition dipole moments and have mutually perpendicular polarizations. This illustrates Davydov splitting [70] of the monomer's light absorption band.

The dozy-chaos–exciton coupling equation for computing optical absorption band shapes in dimers of polymethine dyes can be given approximately [6,7] as

**Figure 9.**
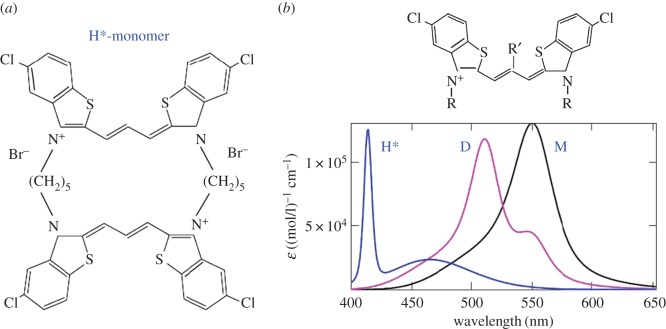
(*a,b*) Theoretical optical absorption band shapes in thiapolymethinecyanines fitted to the available experimental data on polymethine dye monomers (M), dimers (D), and H*-aggregates shown in the general picture presented in 1977 (figure 1*a*) [7]. The polymethine chain length is *L* = 6*d*, where *d* denotes certain, roughly equal bond lengths in the chain. The absorption bands are computed from equations (2.12)–(2.30), (2.34) and (3.1) when fitting them to the experimental data in terms of the wavelength *λ*_max_, extinction coefficient ε_max_ and half-width *w*_1/2_ with a high degree of accuracy. We used the ‘monomer + environment’ system parameters [6] *q* = 1.48*e*, where *e* is the electron charge; *m* = 3.5*m*_e_, *ω* = 5 × 10^13^ s^−1^, *d* = 0.14 nm, *n*_ref_ = 1.33, *J*_1_ = 5.4 eV, *J*_1_−*J*_2_ = 1.42 eV, *E* = 0.21 eV, *γ* = 0.17 eV, and T=298 K; the ‘dimer + environment’ system parameters [6] $\alpha \equiv \alpha (D)=61^{\circ }$, q≡q(D)=1.4e, where *e* is the electron charge; *m* = 3.5*m*_e_, $\omega =5\times 10^{13}\;s^{-1}$, *d* = 0.14 nm, *n*_ref_ = 1.33, *J*_1_ = 5.4 eV, $J_{1}-J_{2}^{-}=1.43$ eV, $J_{1}-J_{2}^{+}=1.56$ eV, hence the excitonic splitting energy $J_{2}^{-}-J_{2}^{+}\equiv \Delta J(D)=0.13$ eV, *E* = 0.21 eV, *γ* = 0.15 eV, and *T* = 298 K; and the ‘H*-aggregate + environment’ system parameters [7] $\alpha \equiv \alpha (H^{*})=70^{\circ }$, $q\equiv q(H^{*})=2.26e$, *m* = 3.5*m*_e_, $\omega =5\times 10^{13}\;s^{-1}$, *d* = 0.14 nm, *n*_ref_ = 1.33, *J*_1_ = 5.4 eV, $J_{1}-J_{2}^{-}=1.67$ eV, $J_{1}-J_{2}^{+}=2.11$ eV, excitonic splitting energy $J_{2}^{-}-J_{2}^{+}\equiv \Delta J(H^{*})=0.44$ eV, *E*^−^ = 0.2 eV, *E* ^+^  = 0.1 eV, $\gamma^{-}=1.16$ eV, $\gamma^{+}=0.09$ eV and *T* = 298 K.

3.1 $$K_{D}=\frac{1}{2}[\left(1+\cos\alpha )K_{+}(\Omega )+(1-\cos\alpha )K_{-}(\Omega \right)],$$

where *α* is the angle between the transition dipole moments of constituent molecules (figure 8); *K*_+_ (*Ω*) and *K*_− _(*Ω*) are the rate constants for the transitions to the respective upper and lower exciton states. These are governed by equations (2.12) and (2.30). An example of the optical absorption band shapes in dimers (D-band) is given, e.g. in figures 1 and 9 [6,7]. We note that in the formation of a dimer from monomers, the arising effect of the exciton shift may be expressed as either a decrease or an increase in the energy gap between the electron-excited and ground states (the theoretical determination of which case is realized for a specific molecular dimer requires quantum-chemical calculations, which are beyond the scope of this article). The scheme of the exciton shift in figure 8, which is usually quoted in the literature, refers to the first case (decrease), while our theoretical results [6,7] in processing the experimental data in figures 1 and 9 relate to the second case (increase). Indeed, in this case, the concurrent consideration of the exciton shift and the exciton splitting must lead with high probability to the close proximity of the maximum of the long-wave wing in the optical band of the dimer and of the maximum in the band of the monomer, which in fact we have in figures 1, 9 and 11 (see below).

### 3.3. Dozy-chaos–exciton coupling in H*-aggregates

The nature of the narrow blue-shifted H*-band (figure 1) is explained by both the presence of dozy chaos and a sufficiently large exciton effect in a quantum transition and, moreover, by the strong interference interaction between them [17]. In the H*-aggregate chromophore (dimer, figure 8), there is a competition between the two exciton transitions (Frenkel exciton) [68–70] through the chaotic reorganization motion of the nuclear environment. As a result, the more intense transition (to the upper exciton state) becomes well organized, which shows itself as a narrow peak in the band shape, and the less intense transition (to the lower exciton state) in contrast becomes more disorganized, which is exhibited by a broad spectral wing (figure 9*b*). Formally, this dynamic interference effect is taken into account by considering the dependence of the dozy-chaos energy *γ* on the exciton states [7].

It is common knowledge that the H*-band is shown by polymethine dyes with a specific structure: H*-aggregates are produced by cyclic bis-thiacarbocyanines, i.e. by two similar polymethine dye monomers lying approximately in the same plane, whose polymethine chains are cross-linked to each other at their ends by hydrocarbon radicals (figure 9*a*) [1]. This linkage results in an essential increase in the exciton coupling, and this in turn causes the above competition between exciton transitions, which elucidates the nature of the H*-band [7]. The caption to figure 9 shows the fitting parameters for the chromophore of the H*-aggregate under the assumption that this chromophore is, as indicated above, the dimer. With this assumption, the shape of the experimental H*-band is well reproduced ( figure 1). We note that the results obtained by V.V.E. on the interpretation of the H*-band [7] are pioneering results in theoretical study of H*-aggregates. Nevertheless, H*-aggregates still remain poorly understood, and we do not currently have any information on, for example, the structure of H*-aggregates, i.e. information on how H*-chromophores (i.e. H*-dimers) pack in H*-aggregates. On the other hand, *a priori* H*-aggregates can be represented by just H*-dimers.

### 3.4. Nature of the broad optical bands in H-aggregates: dozy-chaos–exciton coupling effects from inelastic binary collisions of polymethine species in solutions

**Figure 10.**
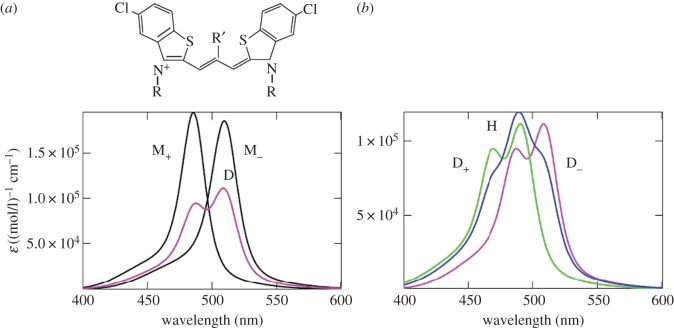
Theoretical optical absorption band shapes in thiapolymethinecyanines fitted to the available experimental data on H-aggregates shown in the general picture presented in 1977 (figure 1*a*). The polymethine chain length is *L* = 6*d*, where *d* denotes certain roughly equal bond lengths in the chain. The absorption H-band (*b*) is computed from equations (2.12)–(2.30), (2.34) and (3.1) when fitting it to the experimental data in terms of the wavelength *λ*_max_, extinction coefficient ε_max_ and half-width *w*_1/2_ with a high degree of accuracy. We used the ‘H-aggregate + environment’ system parameters q≡q(H)=1.51e, $m=3.5m_{e}$, $\omega =5\times 10^{13}\;s^{-1}$, d=0.14 nm, $n_{\mathrm{ref}}=1.33$, $J_{1}=5.4$ eV, $J_{1}-J_{2}^{-}=1.59$ eV, $J_{1}-J_{2}^{+}=1.68$ eV, excitonic splitting energy $J_{2}^{-}-J_{2}^{+}\equiv \Delta J(H)=0.09$ eV, E=0.19 eV, γ=0.13 eV, and T=298 K. In (*a*), for the virtual D-band intermediate dimer angle $\alpha_{M\text{-}M}=104^{o}$ (see the scheme in figure 8), $\varepsilon_{D}(\lambda )=[(1+\cos\alpha_{M\text{-}M})\varepsilon_{M+}(\lambda )+(1-\cos\alpha_{M\text{-}M})\varepsilon_{M-}(\lambda )]/2$. In (*b*), the H-band is produced by a tetramer ($\alpha_{D\text{-}D}=94^{o}$) composed of two intermediate dimers ($\alpha_{M\text{-}M}=104^{o}$) regarded as two novel monomers (supermonomers); $\varepsilon_{D-}(\lambda )=\varepsilon_{D}(\lambda )$ and $\varepsilon_{D+}(\lambda )=\varepsilon_{D}(\lambda +18\;\mathrm{nm})$; $\varepsilon_{H}(\lambda )=[(1+\cos\alpha_{D\text{-}D})\varepsilon_{D+}(\lambda )+(1-\cos\alpha_{D\text{-}D})\varepsilon_{D-}(\lambda )]/2$. The large fitting angles $\alpha_{M\text{-}M}=104^{\circ }$ and $\alpha_{D\text{-}D}=94^{\circ }$ show that H-aggregates are not compact systems.

We now focus on the theoretical absorption band shape for H-aggregates ( figure 1*a*) and on the method for obtaining it theoretically. First, we notice that the structure of the H-band shape differs markedly from that of the D-band (figures 1 and 9). As can be seen from figure 1*a*, the H-band is very wide and has its own peak somewhere in its middle and two feebly marked satellite convexities on either side. This optical band shape may be roughly seen as being composed of two separate band shapes: the band shape for the dimer (D-band) and the band shape symmetrized relative to the peak of D-band with the peaks of these two band shapes coinciding. Therefore, a likely contender for the chromophore of the H-aggregate turns out to be a tetramer. In reality, the H-band is the sum of two D-bands displaced relative to each other and entering it with different weights (see equation (3.1) and figure 10).

It follows from general considerations that the most probable process of producing the chromophore of the H-aggregate (i.e. of the tetramer) in an aqueous solution occurs in two steps. Inelastic binary collisions of monomers initially lead to creating dimers, and inelastic binary collisions of these transient dimers next result in producing tetramers. Based on this, we derive the needed theoretical expression for the optical absorption band shape for H-aggregates in two steps. First, we take the theoretical result for the absorption band shape for dimers (equations (2.12)–(2.30), (2.34), (3.1)) and ‘fit’ it to a probable band shape for transient dimers (figure 10*a*). Then, representing such transient dimers as supermonomers, we once again use the theoretical result for the absorption band shape for dimers (equations (2.12)–(2.30), (2.34), (3.1)), which are now constituted from the novel monomers. By this expedient, we obtain the required theoretical expression for the optical absorption band shape for tetramers (figure 10*b*). During the process, this theoretical result is fitted to the experimental data on the absorption band shape for H-aggregates, and the system parameters are found by trial and error. It is amply clear that the process of such a dual fitting is delicate and expects a certain degree of intellectual skill from the researcher. The resulting angles between the transition dipole moments of the component species are found to be large (see the caption to figure 10). Therefore, in contrast with the compact structure of J-aggregates (brickwork structure; figure 7), the structure of H-aggregates is not the compact pack-of-cards structure, as is usually reported in the literature (e.g. [2,12,13] and the references therein): it is a loose alternate structure. The specific definition of the H-tetramer structure requires additional quantum-chemical calculations, which are beyond the scope of this article. At the same time, the knowledge derived from our assessment of the angles between the transition dipole moments of the component species (*α*_M-M_ = 104° and *α*_D-D_ = 94°; see the caption to figure 10) can significantly reduce the amount of those calculations.

**Figure 11.**
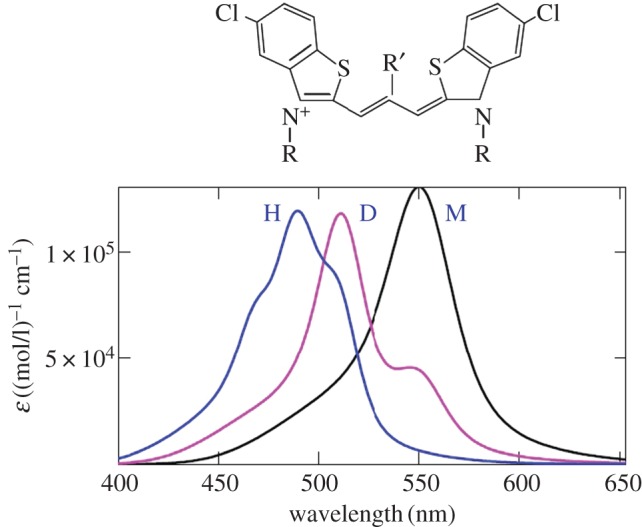
Theoretical optical absorption band shapes in thiapolymethinecyanines fitted to the basic experimental data on polymethine dye monomers (M), dimers (D) and H-aggregates shown in the general picture presented in [2] ( figure 1*a*). The parameters of the ‘species + environment’ systems (monomer and dimer) are given above in the caption to figure 9. The parameters of the ‘H-aggregate + environment’ system are given above in the caption to figure 10. Compare this figure for H-aggregates and figure 9*b* for H*-aggregates.

In figure 11, we give the results of theoretically fitting the optical absorption band shapes for the monomer, dimer and H-aggregate to the experimental data [1,2]. As can be seen from figure 11 and figure 1*a*, there is a good agreement between theory and experiment, especially for the details of the shape of the H-band.

## 4. On a simple method of treating the complex shapes of optical bands and the final results

Using the expressions for optical band shapes for dimers, tetramers, etc., in different combinations in the abovementioned fitting process, we can theoretically elucidate more complicated band shapes, similarly to the band shape for H-aggregates (H-tetramers). Such a design of the optical band shapes for molecular aggregates mirrors the physical process of their production in liquid solutions: aggregates of polymethine dyes are produced in the most probable processes of inelastic multiple binary collisions between polymethine species generally differing in complexity.

**Figure 12.**
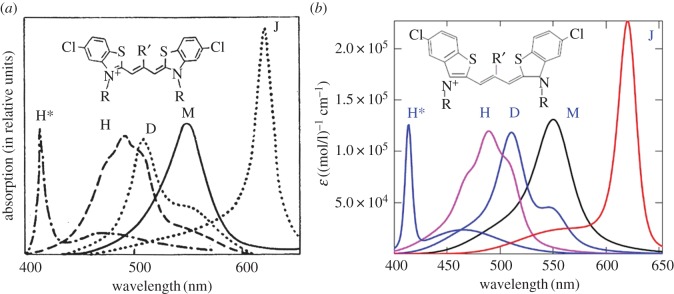
(*a*) Theoretical optical absorption band shapes (*b*) in thiapolymethinecyanines [17] fitted to the basic experimental data (*a*) on polymethine dye monomers (M), dimers (D), H-, H*- and J-aggregates, which are shown in the general picture presented in [2] (figure 1*a*). The fitting parameters for the theoretical band shapes are given in the caption to figure 9 above for the monomer, dimer and H*-aggregate and in the caption to figure 10 above for the H-aggregate. The absorption J-band (*b*) is computed from equations (2.12)–(2.30) and (2.34) when fitting it to the experimental data in terms of the wavelength $\lambda_{\max}$, extinction coefficient $\varepsilon_{\max}$ and half-width *w*_1/2_ with a high degree of accuracy. We used the ‘J-aggregate + environment’ system parameters q≡q(J)=0.97e, $m=1.7m_{e}$, $\omega =5\times 10^{13}\;s^{-1}$, *d* = 0.14 nm, *n*_ref_ = 1.33, *J*_1_ = 5.4 eV, $J_{1}-J_{2}=1.243$ eV, *E* = 0.22 eV, γ = 0.088 eV, and *T* = 298 K.

Finally, in figure 12, we show a comparison of the variety of experimental and theoretical shapes of the optical bands in polymethine dyes (see §1)—monomers, dimers, H-, H*- and J-aggregates.

## 5. Summary and discussions

This work brings some completion to the theoretical explanation of a set of the optical band shapes in polymethine dyes, their dimers and aggregates. The explanation of all this remarkable diversity of optical bands, represented briefly in the final figure (figure 12), is based on a new theory of elementary electronic charge transfers in condensed media, which has been developed by V.V.E. fairly fully, beginning with publications in 2001 [3,4], and in their significantly approximate but still constructive manner [6,19–22], starting with publications in 1988 (although the first results were obtained as early as 1983 and were published in conference proceedings in 1985) [31,32]. This theory is based on rejecting the famous and popular Franck–Condon views [24–27] in the theory of the dynamics of molecular quantum transitions and replacing them with an understanding of the dynamics of the transient state of molecular quantum transitions, which has a chaotic nature [12,13,15,16]. Theoretical analysis of the dynamics of molecular transient states shows that in the process of a quantum transition the motions of electrons and nuclei are not much separated in time, as the Franck–Condon physical picture prescribes; on the contrary, they are aligned in time due to their joint chaotic motion. This chaos in the motion of electrons and nuclei exists only in the transient state and is absent from the initial and final states of molecular systems experiencing quantum transitions. It is therefore called dozy chaos. Introducing dozy chaos into molecular quantum mechanics has a forced nature and is associated with eliminating a substantial singularity in the probabilities (per unit time) of molecular quantum transitions (associated with the incommensurability of electron and nuclear masses), and it is the result of going beyond the adiabatic approximation. Considering a molecular quantum transition in the framework of the adiabatic approximation is tantamount to abandoning the dynamics of the transient state of a molecule, which in any case is always there.

Formally, dozy chaos is introduced into the theory by replacing the infinitesimal imaginary addition in the energy denominator of the total Green's function of a molecular system with a finite value. This procedure was performed in its entirety in the simplest example of molecular quantum transitions taking the dynamics of the transient state into account, namely, in the example of elementary electron-charge transfers in condensed media [3,4]. The simplicity is here associated with the opportunity to approximate the electron Green's function by a propagator and also with the opportunity to consider only non-local phonons and neglect local phonons [4,18]. Because the main optical chromophore of polymethine dyes, the polymethine chain, has a quasi-linear structure with an alternating electronic charge along the chain, which is alternately redistributed on optical excitation, these dyes proved very convenient objects for numerous applications of the new theory of elementary electron-charge transfers. Thus, the theory of the optical band shape in polymethine dyes and their aggregates is not constructed as an *ad hoc* theory, as is often done by physicists (e.g. [12,13] and the references therein) or chemists (e.g. [71] and the references therein), but is constructed as a by-product of the dozy-chaos theory of molecular quantum transitions. Theoretical atomic physics and theoretical nuclear physics were similarly constructed in the twentieth century, for example, as by-products of quantum mechanics.

We briefly dwell on the most basic results of the theory of the optical band shape in polymethine dyes and their aggregates, highlighting the results on H-aggregates to which this article is mainly addressed. It is convenient to divide all the results on the optical band shape into those associated with the transferon resonance (see equation (2.33)) and those not associated with this resonance. We first note that in both cases there are both narrow optical bands, which are of particular interest for applications, and wide optical bands. In the case of polymethine dye monomers, there are two results that are most striking. The first is the result on the theoretical reproduction of the optical band shape in the vinylogous series of thiapolymethinecyanine: the resonant character of the change in the optical band shape with a change in the polymethine chain length (figure 5), obtained by V.V.E. in 2002 [5]. The second striking result is the nature of the change in the entire set of optical bands in the vinylogous series in line with a change in the solvent polarity. When the solvent polarity changes, there is a shift of the transferon resonance ‘along the length of the polymethine chain’ and consequently a change in the sign of the relative intensity of the two bands that are closest to the resonance band (figure 6) [14,58].

In the case of aggregates of polymethine dyes, there are also two results that are most striking: the shapes of narrow optical bands for J- and H*-aggregates. The nature of the narrow red-shifted J-band (figures 1 and 12) is explained by a good dynamic self-organization of the quantum transitions in the chromophore of J-aggregates (four molecules forming a brickwork-type structure; figure 7) for which the environmental nuclear dynamics in weak dozy chaos contributes to the electronic transition in the J-chromophore [3–5,12,13]. In other words, the resonance between the electron motion and the motion of the reorganization of the nuclei environment in the process of molecular quantum transitions (the transferon resonance; see equation (2.33)) is manifested most clearly in J-aggregates. Exciton effects in the shape of the J-band are negligible [12,13]. By contrast, the nature of the narrow blue-shifted H*-band (figures 1 and 12) is explained by both the presence of dozy chaos and a sufficiently large exciton effect in a quantum transition and, moreover, by the strong interference interaction between them [7,17]. In the H*-aggregate chromophore (dimer, figure 8; the constituent monomer is shown in figure 9*a*), the two exciton transitions (Frenkel exciton) [68–70] compete through the chaotic reorganization motion of the nuclear environment. As a result, the more intense transition (to the upper exciton state) becomes well organized, which is manifested as a narrow peak in the band shape, and the less intense transition (to the lower exciton state) in contrast becomes more disorganized, which is exhibited by a broad spectral wing [7,17].

The wide bands of dimers and H-aggregates of polymethine dyes are a result of the non-resonant nature of molecular quantum transitions in conditions of a sufficiently strong dozy chaos and a strong exciton effect. The fact that dimer formation occurs is detected from the reciprocal transformation of the optical absorption band shape in the reversible concentration equilibria of monomers and dimers in solvents by using the law of mass action [6]. All the bands for dimers and monomers at various concentrations intersect at a single isobestic point [6], whose existence indicates that two well-defined components are present only in these concentration equilibria. We note that there are also similar concentration equilibria for the systems of J-aggregates and monomers in solvents [3–5,12,13]. There are no such reversible concentration equilibria for H-aggregates and H*-aggregates. In the case of H-aggregates, the reversible concentration equilibria are absent because H-aggregate production occurs via inelastic collisions of monomers, then dimers, etc. in several stages (in at least two stages; see §3.4). In contrast with the compact brickwork structure of J-aggregates, which have the form of long thin rods [12,13], H-aggregates, showing a large variety of broad optical bands [1], have a loose alternate structure. In the case of H*-aggregates, the absence of the reversible concentration equilibria is apparently explained by the anomalously strong interaction of monomers of cyclic bis-thiacarbocyanines in the H*-dimer [7,17].

The theory of the optical band shape based on dozy chaos and the corresponding calculation scheme for polymethine dyes and their aggregates can, of course, be generalized to a wide range of other objects studied in organic chemistry, but such a generalization, because of its grandeur, will undoubtedly require collective efforts of the scientific community.

On the other hand, for confidence in the reliability of the new physical (dozy-chaos) picture of molecular quantum transitions and to control dozy chaos in the future, its direct detection of what V.V.E. said even earlier in 2013 [6,15,16] is necessary. In this regard, experimentally studying the nuclear dynamics of the loss of regularity of a molecular structure in the chaotic transient state of molecular quantum transitions is one of the most important elements in studying the nature of dozy chaos. This kind of structural dynamics research using X-ray free electron lasers [72] might be one of the key scientific developments in the near future.

## Footnotes

1 The perturbation *V* in equation (2.10) is taken in the usual long-wave approximation (e.g. [30]).

2 The factor ${\bar{n}}_{1}$ arises from taking the equilibrium population distribution for *n*_1_ initial phonon states into account.

3 Heat energy *Δ* < 0 corresponds to the processes inverse to optical absorption, i.e., to luminescence, consideration of which is beyond the scope of this article.

## Data accessibility

Mathcad program files validating the results in figures 1,5,6,9,10–12 are available at the Dryad Digital Repository: http://dx.doi.org/10.5061/dryad.t0r3p [73].
